# Migraine-Associated Common Genetic Variants Confer Greater Risk of Posterior vs. Anterior Circulation Ischemic Stroke

**DOI:** 10.1016/j.jstrokecerebrovasdis.2022.106546

**Published:** 2022-05-13

**Authors:** P. Frid, H. Xu, B.D. Mitchell, M. Drake, J. Wasselius, B. Gaynor, K. Ryan, A.K. Giese, M. Schirmer, K.L. Donahue, R. Irie, M.J.R.J. Bouts, E.C. McIntosh, S.J.T. Mocking, A.V. Dalca, E. Giralt-Steinhauer, L. Holmegaard, K. Jood, J. Roquer, J.W. Cole, P.F. McArdle, J.P. Broderick, J. Jimenez-Conde, C. Jern, B.M. Kissela, D.O. Kleindorfer, R. Lemmens, J.F. Meschia, J. Rosand, T. Rundek, R.L. Sacco, R. Schmidt, P. Sharma, A. Slowik, V. Thijs, D. Woo, B.B. Worrall, S.J. Kittner, J. Petersson, P. Golland, O. Wu, N.S. Rost, A. Lindgren

**Affiliations:** a Department of Clinical Sciences Lund, Neurology, Lund University, Lund, Sweden; b Section of Neurology, Skåne University Hospital, Malmö, Sweden; c Division of Endocrinology, Diabetes and Nutrition, Department of Medicine, University of Maryland School of Medicine, Baltimore, MD, USA; d Geriatric Research and Education Clinical Center, Veterans Administration Medical Center, Baltimore, MD, USA; e Department of Clinical Sciences Lund, Radiology, Lund University, Lund, Sweden; f Department of Neurology, Massachusetts General Hospital, Harvard Medical School, Boston, MA, USA; g Athinoula A. Martinos Center for Biomedical Imaging, Department of Radiology, Massachusetts General Hospital, Harvard Medical School, Charlestown, MA, USA; h Department of Clinical Neuroscience, Institute of Neuroscience and Physiology, Sahlgrenska Academy, University of Gothenburg, Gothenburg, Sweden; i Department of Neurology, Neurovascular Research Group (NEUVAS), IMIM-Hospital del Mar (Institut Hospital del Mar d’Investigacions Mèdiques), Universitat Autonoma de Barcelona, Spain; j Department of Neurology, University of Maryland School of Medicine and Veterans Affairs Maryland Health Care System, Baltimore, MD, USA; k Department of Neurology and Rehabilitation Medicine, University of Cincinnati College of Medicine, Cincinnati, OH, USA; l Department of Laboratory Medicine, Institute of Biomedicine, the Sahlgrenska Academy, University of Gothenburg, Gothenburg, Sweden; m Department of Neurosciences, Experimental Neurology, VIB Center for Brain & Disease Research, Department of Neurology, University Hospitals Leuven, KU Leuven – University of Leuven, Leuven, Belgium; n Department of Neurology, Mayo Clinic, Jacksonville, FL, USA; o Henry and Allison McCance Center for Brain Health Massachusetts General Hospital, Boston, USA; p Department of Neurology, Miller School of Medicine, University of Miami, The Evelyn F. McKnight Brain Institute, FL, USA; q Clinical Division of Neurogeriatrics, Department of Neurology, Medical University Graz, Austria; r Institute of Cardiovascular Research, Royal Holloway University of London (ICR2UL), Egham, United Kingdom; s Department of Neurology, Jagiellonian University Medical College, Krakow, Poland; t Stroke Division, Florey Institute of Neuroscience and Mental Health, and Department of Neurology, Austin Health, Heidelberg, Australia; u Departments of Neurology and Public Health Sciences, University of Virginia, Charlottesville, VA, USA; v Computer Science and Artificial Intelligence Laboratory, MIT, Cambridge, USA; w Section of Neurology, Skåne University Hospital, Lund, Sweden; x Department of Radiology, Neuroradiology, Skåne University Hospital, Lund, Sweden; y Department of Neurology, Sahlgrenska University Hospital, Gothenburg, Sweden; z Department of Clinical Genetics and Genomics, Sahlgrenska University Hospital, Gothenburg, Sweden

**Keywords:** Posterior circulation ischemic stroke, MRI phenotype, Migraine, Common genetic variants

## Abstract

**Objective:**

To examine potential genetic relationships between migraine and the two distinct phenotypes posterior circulation ischemic stroke (PCiS) and anterior circulation ischemic stroke (ACiS), we generated migraine polygenic risk scores (PRSs) and compared these between PCiS and ACiS, and separately vs. non-stroke control subjects.

**Methods:**

Acute ischemic stroke cases were classified as PCiS or ACiS based on lesion location on diffusion-weighted MRI. Exclusion criteria were lesions in both vascular territories or uncertain territory; supratentorial PCiS with ipsilateral fetal posterior cerebral artery; and cases with atrial fibrillation. We generated migraine PRS for three migraine phenotypes (*any migraine; migraine without aura; migraine with aura*) using publicly available GWAS data and compared mean PRSs separately for PCiS and ACiS vs. non-stroke control subjects, and between each stroke phenotype.

**Results:**

Our primary analyses included 464 PCiS and 1079 ACiS patients with genetic European ancestry. Compared to non-stroke control subjects (*n*=15396), PRSs of *any migraine* were associated with increased risk of PCiS (*p*=0.01–0.03) and decreased risk of ACiS (*p*=0.010–0.039). *Migraine without aura* PRSs were significantly associated with PCiS (*p*=0.008–0.028), but not with ACiS. When comparing PCiS *vs*. ACiS directly, migraine PRSs were higher in PCiS *vs*. ACiS for *any migraine* (*p*=0.001–0.010) and *migraine without aura* (*p*=0.032–0.048). *Migraine with aura* PRS did not show a differential association in our analyses.

**Conclusions:**

Our results suggest a stronger genetic overlap between unspecified migraine and migraine without aura with PCiS compared to ACiS. Possible shared mechanisms include dysregulation of cerebral vessel endothelial function.

## Introduction

Migraine, particularly with aura, confers an increased risk of cardiovascular events and ischemic stroke.^1–4^ Manifestations of cerebrovascular disease including subclinical ischemic lesions and white matter abnormalities on magnetic resonance imaging (MRI) are more common in migraineurs than in control subjects,^5,6^ though this is not a consistent finding.^7^ Several studies report that ischemic and infarct-like lesions in migraine patients predominate in the posterior circulation territory but the reasons for these observations are not clear.^8–10^

A previous study revealed several genetic associations between ischemic stroke, including specific etiologies, and migraine phenotypic traits.^11^ However, posterior circulation ischemic stroke (PCiS) has not been explored as a distinct phenotype in relation to migraine in genetic association studies. The above observations provide a biological rationale for exploring a possible shared genetic background between migraine and PCiS vs. anterior circulation ischemic stroke (ACiS). To evaluate the relation of migraine with ischemic stroke topography, we used a target population of ischemic stroke patients with MRI verified acute ischemic lesions phenotyped as PCiS or ACiS based on lesion localization on diffusion weighted imaging (DWI).^12^ We developed polygenic risk scores (PRSs) from previously published migraine genome wide association study (GWAS) data^13^ to address two complementary questions. First, we tested if the associations of migraine PRSs with stroke were stronger for PCiS than for ACiS. Second, we tested whether genetic predisposition to migraine was associated with increased risk of posterior or anterior circulation ischemic stroke.

## Materials and methods

### Ischemic stroke population

Ischemic stroke cases were collected from the neuroimaging repository of the MRI-GENetics Interface Exploration (MRI-GENIE) collaboration.^14^ Data in MRI-GENIE have been provided by 12 of the National Institute of Neurological Disorders and Stroke Genetics Network (SiGN) study sites.^15^ Previous publications in detail describe the SiGN and MRI-GENIE studies.^14,16^ We reviewed all MRI-DWI images and phenotyped them as PCiS or ACiS according to criteria previously published.^12^ Phenotyping was done blinded to genotype data. The MRI-GENIE cohort contained 718 PCiS and 1663 ACiS patients. In this study, we excluded patients with DWI lesions in the posterior cerebral artery territory with a concomitant ipsilateral fetal posterior cerebral artery. We also excluded patients with atrial fibrillation because distal emboli were anticipated to be proportionally equally distributed between the posterior and anterior circulation territories. We stratified our analyses based on ancestry and genotyping platforms. The remaining number of patients for the genetic analyses was 505 with PCiS and 1182 with ACiS (Table 1).

### Ischemic stroke cohort genotyping

Genome-wide genotyping data were available for all participants in the MRI-GENIE cohort through SiGN. Patients were either genotyped through the enrollment site and their genotype data subsequently submitted to SiGN, or within SiGN at the Center for Inherited Disease Research (CIDR).^16^ Previous publications detail the platform characteristics and the genotyping data analysis strategy and quality control methods within SiGN/MRI-GENIE.^14,16^ In our target population a majority of cases, 433 (86%) with PCiS, and 997 (84%) with ACiS were genotyped at CIDR on the Illumina HumanOmni5Exome-4v1 platform. For the main analyses, we used ischemic stroke cases of European ancestry (EUR) only (n=1543). In the transethnic analyses, we also included cases of African ancestry (AFR) (n=144).

#### Imputation and post-imputation quality control (QC)

SiGN genotype data were previously cleaned.^17^ For the current analyses, we re-imputed the SiGN genotype data with the TOPMed multiethnic reference panel using Michigan Imputation Server.^18^ Minimac4 Algorithm was used for the imputation. The imputed genotype data were based on human genome build hg38. Imputed genetic variants with imputation quality score R^2^<0.5 were excluded from further analyses.

### Non-stroke control subjects

Genotype data from 15 396 non-stroke control subjects were available through SiGN. In SiGN, the control subjects were comparable to stroke cases in terms of ancestry and genotyping array. Age data were not available for all control subjects. Extensive QC has been conducted and documented by SiGN.^15^

### Migraine GWAS data

We used published migraine GWAS summary results^13^ for the phenotypes any migraine (M), migraine without aura (MO) and migraine with aura (MA) to generate individual PRSs in MRI-GENIE cases with PCiS and ACiS. The previously published migraine GWAS was a meta-analysis of 22 migraine GWAS studies of 375 000 individuals with 59 674 cases and 316 078 control subjects.^13^ Migraine classification in the studies used for the discovery meta-analysis was largely based on a combination of the International Classification of Headache Disorder II criteria (ICHD II)and self-reporting.^13^ The number of cases for each phenotype were: M 59 674; MO 8348; and MA 6 332. All samples in the migraine GWAS were of European genetic ancestry.^13^

### Polygenic risk scores

The generated individual level PRSs for PCiS and ACiS cases were used to calculate 3 or 4 *p*-value cutoffs (between 10^−5^ and ≤10^−8^) for all discovery phenotypes. MA scores did not include the ≤10^−8^ level because there were no genome-wide associations detected for MA at this significance level in the migraine GWAS data.

We used the PRSice-2^19^ software package to calculate PRS for the MRI-GENIE study individuals. The migraine-associated Single Nucleotide Variants (SNVs) included in the summary statistics meeting each p-value threshold were clumped to remove SNVs in linkage disequilibrium (LD) with each other. We retained only the SNVs with the lowest GWAS p-value in each LD block. The allelic dosages for each SNV were weighted by their beta (β) coefficients from the migraine summary statistics GWAS. The weighted beta coefficients were then summed across all SNVs to generate individual PRSs, i.e.

$$\text{PolygenicRiskScore}\;(\mathrm{PRS})=\sum [(\beta_{i}*\text{ADs})/\text{Mj}]$$

where ${\text{β}}_{i}$ indicates β value for the *i*_th_ SNV from the migraine GWAS summary statistics and ADs = allelic dosage for each case. M is the number of alleles included in the PRS of the individual. To test the specificity of the migraine PRSs’ association with PCiS *vs*. ACiS, we performed secondary analyses in which the association of the migraine PRSs was compared with non-stroke control subjects for PCiS and ACiS separately.

### Data availability

Access to anonymized clinical and genetic data related to this study will be made available on request and may be subject to material transfer agreements. Requests for imaging and clinical data relating to the MRI-GENIE study can be made to NSR.

### Statistical analyses

In our primary analyses, we tested whether genetic risk of migraine was more strongly associated with PCiS than with ACiS by comparing migraine PRSs for three migraine traits between PCiS and ACiS in European ancestry cases. To further test whether genetic risk of migraine was differentially associated with PCiS or ACiS, we performed secondary analyses comparing migraine PRS between PCiS and ACiS and non-stroke control subjects separately. We also performed analyses by using the two approaches above and including cases of African ancestry in the target group. We conducted logistic regression analyses for the primary analysis and the secondary analyses, controlling for age, sex and principal components (PC) of genetic ancestries (PC 1-10). Analyses were stratified by genetic ancestry groups and genotyping platforms, as described in the SiGN meta-analysis publication.^15^ The results of the logistic regression analysis for each stratum were meta-analyzed using an inverse variance weighting based method, assuming fixed effects. Our primary analyses comparing migraine PRS in PCiS with ACiS were based on EUR cases (n=464 PCiS, n=1079 ACiS) because the migraine GWAS included only EUR cases. In the primary analyses we applied Bonferroni correction for multiple testing, considering a p-value of 0.016 to be significant, based on analyzing three migraine traits. This correction is stricter than necessary since migraine traits were not completely independent from each other. The method of using PRSs based on different GWAS p-value thresholds was not corrected for multiple testing due to the complete overlapping sets of SNPs from one threshold to the next. In the meta-analyses combining EUR and AFR cases (n=505 PCiS, n=1182 ACiS) we assumed first fixed then random effect. For all analyses, estimated beta coefficients from the logistic regression analyses were converted to odd ratios and expressed as per one standard deviation increases of the PRSs. Post-hoc power calculations were not performed since significant p-value results were for the PCiS group despite our study’s higher power for the ACiS group.

### Standard protocol approvals, registrations and patient consents

All study protocols regarding human subjects have been approved by their local institutional review board, and written consent was given by all participants or through surrogate authorization at the time of enrollment at the original sites. The MRI-GENIE study has been approved by the institutional review board at the Massachusetts General Hospital, Boston, MA (protocol number: 2001P001186).

## Results

There were a total of 8021 SNVs reaching p-values of ≤10^−5^ in the migraine GWAS meta-analysis. The number of SNVs included for each migraine phenotype were 7208 (M), 1173 (MO), and 464 (MA).

### Primary analyses

#### Migraine PRSs in PCiS *vs*. ACiS subjects: analysis in EUR samples

**Fig. 1.**
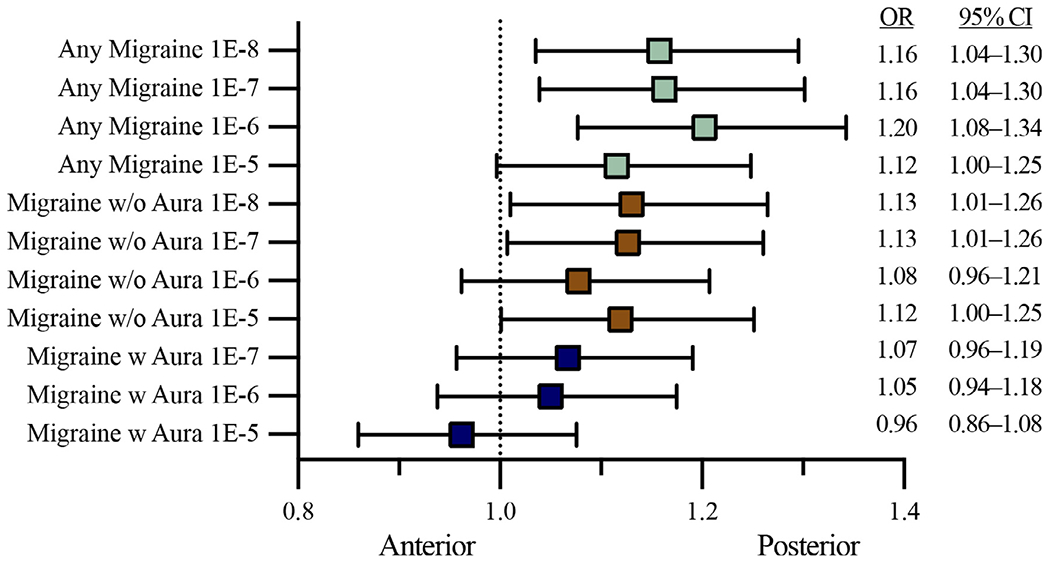
*Migraine PRSs and risk of posterior* vs. *anterior circulation ischemic stroke. Estimated beta coefficients converted to odds ratios and expressed as per one standard deviation increases of the PRS. PRS, Polygenic risk score; w/o, without; w, with*

We performed meta-analyses of migraine mean PRSs for PCiS subjects vs. ACiS subjects for the three migraine phenotypes (Fig. 1) and considered Bonferroni corrected p ≤ 0.016 to be significant.

##### Any migraine (M)

PRSs for the *M* phenotype across GWAS *p*-value thresholds from 10^−6^ to 10^−8^ were significantly higher in PCiS compared to ACiS (*p*-range between 0.001 and 0.010, OR-range between 1.16 and 1.20). The corresponding difference using the PRS of *M* constructed from SNVs with GWAS *p*-values ≤10^−5^ remained consistent (OR=1.12) but with borderline statistical significance (*p*=0.057) before Bonferroni correction.

##### Migraine without aura (MO)

PRSs for the *MO* phenotype were significantly higher in PCiS than in ACiS at the cutoff levels of ≤10^−5^, ≤10^−7^ and ≤10^−8^ with non-corrected association p-values of 0.032–0.048 (OR =1.25–1.26), which is nominally significant (*p*<0.05) but do not meet the strict Bonferroni corrected p-value cutoff (*p*<0.016).

##### Migraine with aura (MA)

*MA* derived PRSs did not significantly differ between the two stroke phenotypes at any cutoff level.

### Secondary analyses

#### Migraine PRSs in PCiS and ACiS separately *vs*. non-stroke control subjects: analyses in EUR samples

The association of migraine PRSs were compared with non-stroke control subjects for PCiS and ACiS separately (Table 2).

##### Any migraine (M)

PRSs for the *M* phenotype were associated with increased risk of PCiS (OR=1.13–1.15, *p*=0.011–0.03 for PRS at GWAS *p* thresholds ≤10^−5^, ≤10^−6^ and ≤10^−7^) but decreased risk of ACiS (OR=0.91–0.93, *p*=0.010–0.039 for PRSs at GWAS *p* thresholds ≤10^−6^, and ≤10^−7^).

##### Migraine without aura (MO)

PRSs for the *MO* phenotype were significantly associated with PCiS compared with non-stroke control subjects with association p-values of 0.008 and 0.028 (OR=1.12–1.15), respectively for the PRSs at GWAS *p*-value thresholds ≤10^−5^ and ≤10^−7^. There was no significant association between *MO* PRSs and the risk of ACiS compared with non-stroke control subjects.

##### Migraine with aura (MA)

PRSs for *MA* showed no significant association with the risk of PCiS or ACiS at any GWAS *p*-value cutoff level when compared to non-stroke control subjects.

#### Migraine PRSs in PCiS *vs*. ACiS in EUR and AFR samples

In the transethnic meta-analysis of migraine PRS in PCiS *vs*. ACiS, PRS associations with PCiS for the *M* phenotype remained significant (*p*=0.001–0.012) compared with ACiS for various SNV selection criteria (migraine GWAS *p*-value cutoff ≤10^−6^–≤10^−8^). Even though only borderline significance was observed for the same PRS *p*-value cutoff range (≤10^−6^–≤10^−8^) for higher *MO* PRS in PCiS compared to ACiS (*p*=0.055–0.089), the effect size and direction remained similar (OR=1.24). Meta-analysis assuming random effect did not significantly alter the results compared to assuming fixed effect.

#### Migraine PRSs in PCiS and ACiS separately *vs*. non-stroke control subjects in EUR and AFR samples

##### Any migraine (M)

The association results were similar for PRSs at the GWAS p-value threshold ≤10^−6^ compared with the analysis for EUR cases. PRSs of *M* showed a significant association with increased risk of PCiS, but decreased risk of ACiS at the cutoff level of ≤10^−6^ only. However, at other GWAS p-value thresholds, the PRSs for any migraine did not show association with either ischemic stroke phenotype when compared separately to non-stroke control subjects.

##### Migraine without aura (MO)

The association between *MO* PRSs and the risk of PCiS was even more prominent in the EUR+AFR analysis; PRSs for the *MO* phenotype were significantly associated with the risk of PCiS at all GWAS p thresholds (≤10^−5^–≤10^−8^, *p*-values 0.012–0.038, OR=1.10–1.13). There was no significant association between *MO* PRS and ACiS risk compared with non-stroke control subjects.

##### Migraine with aura (MA)

Consistent with EUR only analysis, PRSs for *MA* showed no significant association with PCiS or ACiS when compared to non-stroke control subjects at any cutoff level for the polygenic score.

The results of the transethnic analyses of PCiS and ACiS vs. non-stroke control subjects are presented in Table 3.

## Discussion

We present PRS data indicating a shared genetic contribution to the risk of migraine and migraine without aura and posterior circulation ischemic stroke. Genetic predisposition to migraine in this study was more related to PCiS than to ACiS, possibly indicating separate genetic predisposition or biological pathways in PCiS vs. ACIS. Our results implicate only *M* PRSs and *MO* PRSs with the risk of PCiS, because *MA* PRSs showed no significant association with either stroke phenotype. In contrast, a recent GWAS^11^ showed significant association of *MA* derived PRSs with all stroke and the subtype large artery stroke. However, that study also demonstrated a stronger genetic overlap overall for *MO* and ischemic stroke compared with *MA*. The weaker genetic association between *MA* and ischemic stroke in GWAS and in our study – as opposed to epidemiological data suggesting a stronger association between *MA* and ischemic stroke – could be partially explained by rare genetic variants not revealed through GWAS, since such rarer variants may be more dominant genetic factors in *MA*.^20^ The discrepancy between previous results^11^ and our study may thus be related to the smaller number of *MA* related SNVs discovered through GWAS (465) compared with *MO* (1 174), and the comparatively low number of ischemic stroke cases in our study. We did not perform stratified analyses for ischemic stroke subtypes in relation to migraine PRSs because of the small sample sizes for the respective subtypes, which would result in insufficient power to detect differences in the strength of association with migraine PRSs.

Neuroimaging studies have reported an increased prevalence of ischemic and infarct-like lesions and white matter hyperintensities in migraine patients.^6,21^ Such findings are more frequent in the posterior vascular territory, mainly in female *MA* patients and in high attack frequency.^5,22^ A few studies have reported a higher burden of infarct-like lesions in the cerebellum than in other locations; in female migraineurs with aura compared to control subjects,^21^ and in unselected migraine patients with or without aura compared to control subjects.^23^ The influence of sex on the results could not be addressed in our study due to the relatively small sample sizes in the target cohort. It is interesting to note that male sex has been reported to be an independent risk factor for PCiS compared with ACiS.^12,24^ Conversely, the prevalence of migraine headache is higher in females,^25^ but during a different age span than when ischemic stroke typically occurs.

Studies investigating cerebral vascular reactivity as a marker of endothelial function have demonstrated differences between the posterior and anterior circulation in migraine patients,^26–29^ whereas studies on systemic endothelial dysfunction as a contributory mechanism have been contradictory.^30,31^ This suggests a possible link regarding endotheliopathy related to vascular brain changes in the posterior circulation territory and migraine. Endothelial cell signaling pathways and vascular smooth muscle function have been implicated in migraine pathophysiology. We speculate that endothelial properties in the vertebrobasilar arteries may contribute to atherogenesis and thrombus formation through biological mechanisms that are unclear, thus increasing the risk of PCiS in predisposed individuals. The two largest GWAS studies of migraine to date have identified several loci linked to genes associated with vascular and smooth muscle function.^13,32^ Clincal, genetic and neuroimaging data, including the findings in our present study thus suggest that it is reasonable to conceptualize PCiS as a distinct phenotype in relation to migraine. The benefit of MRI defined ischemic stroke phenotypes was recently highlighted in a pooled analysis of GWAS and individual data of lacunar stroke in which genetic associations and heritability measures were stronger in MRI defined lacunar strokes than previous analyses based on TOAST.^33^

The genetic ancestry homogeneity in this study, and in the GWAS field generally, warrants mentioning. The samples in the migraine GWAS meta-analysis on which our PRS calculations are based were of European ancestry only,^13^ but chosen for the much larger sample size compared to migraine GWASs with more diverse populations. Our target group also mainly included European ancestry cases. Hence, we used European ancestry samples in the main analyses to maximize the accuracy and power of the PRS method. When including target cases of African ancestry, the results for *M* PRSs remained similar, while *MO* PRSs only reached borderline significance despite similar effect sizes and directions. The difference is likely due to the fact that the migraine GWAS was conducted in EUR only cases, and the European based migraine PRS has suboptimal performance for African ancestry cases, limiting generalizability in African ancestry populations.^34^ The sample size of the African ancestry stratum was also small (n=144). Future studies with more diverse ancestries in migraine GWAS and in the stroke cohorts are needed.

### Strengths

The image based phenotyping of our ischemic stroke target group represents a major strength of this study. All included patients had acute ischemic lesions on MRI-DWI, and were grouped according to vascular territory of lesion (anterior/posterior). These image-based stroke cases represent enriched phenotypes, minimizing the influence of bias such as diagnosis based only on clinical assessment and medical record review. The definitive diagnosis of lesions of vascular origin in our target group strengthens the results. The inclusion of non-stroke control subjects in addition to the direct comparison of PCiS *vs*. ACiS adds further strength to the findings of the primary analysis. The PRS method represents a strength compared to epidemiological methods, which are more sensitive to confounding factors introduced by environmental influences, such as life style choices and medication usage.

### Limitations

There are some limitations in our study. Only patients investigated with MRI and with visible DWI lesions were included in our target cohort, leading to selection bias in terms of stroke severity if certain patients were not considered for MRI investigation. Migraine comorbidity data in the ischemic stroke cohort were unavailable, preventing determination of phenotypic association between migraine and stroke directly to support the conclusion of our current findings. The number of ischemic stroke cases was small which may have caused insufficient power to detect subtle association. However, the migraine PRS association with PCiS and not ACiS was consistent across several cutoff levels and also remained when comparing the stroke phenotypes separately to a large group of non-stroke control subjects, strengthening our observation of a differential association. We acknowledge that in this exploratory study, our sample size precludes stringent correction for multiple testing. Still, in the primary analysis in which correction was applied, a significant difference in PRS association remained.

## Conclusion

We contribute findings suggesting a genetic overlap in migraine and PCiS, specifically *vs*. ACiS. Underlying mechanism may be shared dysfunction in vascular regulatory mechanisms and endothelial cell signaling. The findings indicate that there may be a specific genetic contribution to the risk of PCiS as compared to ACiS. We find the results across strata and in comparison with non-stroke control subjects sufficiently consistent to merit replication in studies with larger sample sizes of ischemic stroke patients with these neuroimaging subtypes.

## Figures and Tables

**Table 1.** Ischemic stroke patient characteristics.

	PCiS n = 505 (%)	ACiS n = 1 182 (%)	*p*-value
Age (mean, SD)	60.9±14.5	63.2±14.5	0.003^a^
Male	342 (67.7)	721 (61.0)	0.009^b^
Diabetes mellitus	133 (26.4)	265 (22.4)	0.081^b^
Hypertension	313 (62.0)	772 (65.3)	0.207^b^
CAD	74 (14.6)	186 (15.7)	0.555^b^
CCS (5 item)			<0.001^b^
CE	21 (4.0)	73 (6.2)	
LAA	106 (21.0)	338 (32.8)	
Other	53 (10.5)	77 (6.5)	
SAO	116 (23.0)	188 (15.9)	
Undetermined	209 (41.4)	506 (42.8)	
Genetic Ancestry			0.307^b^
European	464 (91.9)	1 079 (91.3)	
African American	41 (8.1)	103 (8.7)	

PCiS, posterior circulation ischemic stroke; ACiS, anterior circulation ischemic stroke; CAD, coronary artery disease; CCS, causative classification of stroke; CE, cardioembolic (patients with AF not included in this study); LAA, larger artery atherosclerosis; SAO, small artery occlusion

a T-test

b Chi-square test

**Table 2.** Migraine PRS in PCiS and ACiS separately vs. non-stroke control subjects: EUR samples.

Migraine phenotype	GWAS p cutoff	PCiS vs. non-stroke control subjects			ACiS vs. non-stroke control subjects		
		*p*-value	OR	95% CI	*p*-value	OR	95%CI
Any Migraine	1 × 10^−8^	0.081	1.10	0.99–1.22	0.064	0.94	0.87–1.00
	1 × 10^−7^	*0.030*	1.13	1.01–1.25	*0.039*	0.93	0.86–1.00
	1 × 10^−6^	*0.011*	1.15	1.03–1.28	*0.010*	0.91	0.85–0.98
	1 × 10^−5^	*0.023*	1.13	1.02–1.26	0.440	0.97	0.91–1.04
Migraine without Aura	1 × 10^−8^	0.069	1.10	0.99–1.23	0.913	1.00	0.94–1.08
	1 × 10^−7^	*0.028*	1.12	1.01–1.25	0.381	1.03	0.96–1.11
	1 × 10^−6^	0.091	1.10	0.99–1.22	0.348	1.03	0.96–1.11
	1 × 10^−5^	*0.008*	1.15	1.04–1.28	0.600	1.02	0.95–1.09
Migraine with Aura	1 × 10^−7^	0.212	1.07	0.96–1.18	0.514	1.03	0.95–1.11
	1 × 10^−6^	0.152	1.08	0.97–1.19	0.271	1.04	0.97–1.12
	1 × 10^−5^	0.916	1.01	0.91–1.12	0.051	1.07	1.00–1.15

PRS, polygenic risk score; PCiS, posterior circulation ischemic stroke; ACiS, anterior circulation ischemic stroke; EUR; European ancestry; GWAS, genome-wide association study

**Table 3.** Migraine PRS in PCiS and ACiS vs. non-stroke control subjects: EUR and AFR samples.

Migraine phenotype	GWAS *p* cutoff	PCiS vs. non-stroke control subjects			ACiS vs. non-stroke control subjects		
		*p*-value	OR	95% CI	*p*-value	OR	95%CI
Any Migraine	1 × 10^−8^	0.153	1.07	0.97–1.18	0.156	0.95	0.89–1.02
	1 × 10^−7^	0.091	1.09	0.99–1.20	0.066	0.94	0.88–1.00
	1 × 10^−6^	*0.030*	1.11	1.01–1.22	*0.010*	0.92	0.86–0.98
	1 × 10^−5^	0.091	1.09	0.99–1.20	0.291	0.97	0.91–1.03
Migraine without Aura	1 × 10^−8^	*0.026*	1.12	1.01–1.23	0.488	1.02	0.96–1.09
	1 × 10^−7^	*0.012*	1.13	1.03–1.24	0.188	1.04	0.98–1.11
	1 × 10^−6^	*0.038*	1.10	1.01–1.21	0.162	1.05	0.98–1.11
	1 × 10^−5^	*0.015*	1.13	1.02–1.25	0.642	1.02	0.95–1.09
Migraine with Aura	1 × 10^−7^	0.212	1.07	0.96–1.18	0.443	1.03	0.96–1.11
	1 × 10^−6^	0.249	1.06	0.96–1.17	0.416	1.03	0.96–1.10
	1 × 10^−5^	0.792	1.01	0.92–1.12	0.115	1.05	0.99–1.13

PRS, polygenic risk score; PCiS, posterior circulation ischemic stroke; ACiS, anterior circulation ischemic stroke; EUR, European ancestry; AFR, African ancestry
